# Modification and Validation of an Autism Observational Assessment Including ADOS-2^®^ for Use with Children with Visual Impairment

**DOI:** 10.1007/s10803-024-06514-z

**Published:** 2024-09-09

**Authors:** Naomi Dale, Elena Sakkalou, Maria H. Eriksson, Alison Salt

**Affiliations:** 1 Great Ormond Street Hospital NHS Trust, Great Ormond Street, London, WC1 3JH UK; 2 UCL Great Ormond Street Institute of Child Health, London, UK; 3 Anglia Ruskin University Cambridge, Cambridge, UK

**Keywords:** Social communication, Autism, Autism Spectrum disorder, Visual impairment, Blindness, Child, Assessment, Diagnosis, Play based assessment

## Abstract

Children with visual impairment (VI) are at risk of autism spectrum disorder (ASD); however standard observational diagnostic assessments are not validated for this population. The primary objective of the study is to validate a modified version of the Autism Diagnostic Observation Schedule (ADOS-2^®^, Module 3), for children with VI. A cross-sectional observational study was undertaken with 100 (mean 5½ years, SD 10.44 months, range 4–7 years; 59 (59%) males) children with congenital disorders of the peripheral visual system with moderate/severe-profound VI. As the primary objective, 83 (83%) who were ‘verbally fluent’ were assessed with the modified ADOS-2^®^ (Module 3). Their scores were investigated for reliability, construct and criterion validity against expert clinician formulation and parent-rated social and communication questionnaires (Social Responsiveness Scale-2, SRS-2; Children’s Communication Checklist-2). The secondary objective with the total sample was to report on frequency and distribution of ASD ratings in this VI population. The modified ADOS-2^®^ (Module 3) was found to have strong internal coherence and construct validity (two factor model) and inter-rater reliability. A new VI diagnostic algorithm was established which showed high sensitivity and specificity against clinician formulation. Using the best cut-off threshold for ‘High Risk for ASD’, strong concurrent criterion validity was found according to parent-rated scores on the SRS-2. The modified ADOS-2^®^ (Module 3) was shown to have promising reliability and validity in establishing children at ‘High Risk of ASD’ in this vulnerable population. Elevated rates of ASD were found across the sample, in line with previous estimates.

**Supplementary Information:**

The online version contains supplementary material available at 10.1007/s10803-024-06514-z.

Congenital vision disorders with severe visual impairment (VI) are low incident conditions with significant long-term development, learning and social participatory implications. Prevalence estimates are 5 per 10,000 with moderate-severe VI/blindness in first year of life, with a cumulative incidence of 10/10,000 in children under 18 in the UK (Teoh et al., 2021) and global estimates of 1.4 million children who are blind (Solebo et al., 2017) Their aetiologies are heterogeneous; in higher income countries about half arise from congenital disorders of the peripheral visual system (i.e. disorders of the globe, retina and anterior optic nerve) – often of very rare genetic origin, and half from cerebral disorders including pre-, peri- and early post-natal brain injury and cortical and subcortical visual processing pathway dysfunctions (Sonksen & Dale, 2002; Teoh et al., 2021).

Lack of sensory access to the visuo-social environment delays or prevents learning and development of mutual eye gaze, facial affect expressions, non-verbal imitation, preverbal and gestural communication, social referencing or joint attention, attention-shifting between object and person, imaginative symbolic play, aspects of semantic concepts or pragmatic language use, theory of mind and perspective-taking and affective reciprocal adult-child and peer social relationships (Bathelt et al., 2017; Brown et al., 1997; Dale & Salt, 2008; Dale et al., 2014; Green et al., 2004; Minter et al., 1998; Pring et al., 1998; Parr et al., 2010; Pijnacker et al., 2012; Tadic et al., 2010). Although some of these challenges improve or are circumvented through non-visual (e.g. audition, touch and oral language) or visual compensatory strategies (e.g. augmentative visual aids) as the child learns and matures, some children continue to show persistent social and behavioural features in line with the Autism Spectrum Disorder (ASD) classification (APA 2013). Research studies using a variety of different measures have identified marked ASD-related features (including social interaction, communication and language, restricted behaviours and interests) across the preschool and school years (Absoud et al., 2011; Jutley-Neilson et al., 2013; Jure et al., 2016; Mukaddes et al., 2007). Emerging autistic symptomatology including sustained developmental plateauing or regression (‘developmental setback’) is found in at least a third of children with profound VI and can be differentiated as early as the second to third year of life (Cass et al., 1994; Dale & Sonksen, 2002).

A recent systematic review and meta-analysis estimated that the overall prevalence of ASD in VI and Hearing impairment (HI) populations was 19% (95% CI 13–25%) and 9% (95% CI 6–12%) respectively. The overall risk-ratio of ASD was 31.0 times (95% CI 18.6–51.6); z = 13.2, *p* < 0.001) in the VI population and 14.1 times in the HI population compared to ASD prevalence in the general population (Do et al., 2017). The authors concluded that there is a particularly high association between ASD and VI and therefore expert assessment for ASD is required if there are concerns. Rates of early preverbal autism symptomatology (as in ‘developmental setback’) appear highest in those with additional intellectual and neurological complexity and more severe rates of VI, particularly profound VI (light perception at best) (Dale & Sonksen, 2002; Dale et al., 2019; Mukkades et al., 2007; Parr et al., 2010). Small-scale exploratory studies suggest higher rates of weaker socio-communicative characteristics consistent with the broader autism spectrum in those with verbal cognition and expressive language ability in the average to superior range (Tadic et al., 2010, Bathelt et al., 2017; Greenaway et al., 2017). These difficulties included challenges with peer relationships, emotional regulation, use of context in conversation, adaptive behaviour and stereotyped language.

As in other populations at high risk of ASD, there is a clinical need to diagnose social communicative risks including ASD in a reliable manner. Current standardised ‘gold standard’ measures incorporate ratings of vision-dependent behaviours (such as eye gaze, facial expression, referential joint attention and integration of non-verbal communication and language) which are inappropriate for VI. Communication and behaviour presentations may differ in pattern and intention to those of typically sighted children, making them harder to interpret and scoring more challenging and less reliable (Absoud et al., 2011; Ludwig et al., 2022; Molinaro et al., 2020). The current Autism Diagnostic Observation Schedule, 2nd Edition (ADOS-2^®^) and its scientific diagnostic algorithms are not intended for and have not been validated for children with VI (Lord et al., 2012). Pilot small-scale and some larger studies with children with VI (Butchart et al., 2017; Fazzi et al., 2019; Jure et al., 2016; Mukkades et al., 2007; Williams et al., 2014) have attempted to use semi-adapted versions of existing tools such as the Child Behaviour Checklist (Achenbach & Rescorla, 2000), ADOS-2 (Lord et al., 2012), and Childhood Autism Rating Scale (CARS-2, Schopler et al., 1980) with removal of some vision dependent items. These do not reach stringent psychometric standards and lack scientific validation (Ludwig et al., 2022; Molinaro et al., 2020; Stevenson & Tedone, 2024). A preliminary exploratory validation of a VI-specific observational schedule, the Visual Impairment Social communication Schedule (VISS), with a consecutive sample of pre-schoolers with severe VI, found a high construct validity with CARS ratings and a positive predictive value of 100% in predicting a later clinical diagnosis of autism at 5–6 years (Absoud et al., 2011).

Diagnostic differentiation of ASD in children with VI is more complex and unreliable during the early years. Congenital VI impacts adversely on all areas of development with delays and difficulties in sensorimotor understanding, expressive and comprehension language and social and behavioural development in the infant and preschool years, and with greatest delays in those with profound VI (Dale et al., 2014, 2017; Reynell & Zinkin, 1975; Vervloed et al., 2000; Veldhorst et al., 2022). If language and cognition are progressing quite steadily, it may not be feasible or reliable to establish a longstanding ASD, affecting social, communication and behavioural development, until around 5–6 years or older (Dale & Salt, 2022). Due to their potentially atypical developmental trajectories in the first three to four years of life, diagnostic history-taking through instruments like the Autism Diagnostic Interview Revised (ADI – R, Rutter et al., 2003) may fail to reliably differentiate between those who are at significant risk of ASD or those who are affected mainly by their lack of vision.

To advance in the clinical imperative of establishing a reliable means of assessing and identifying ASD in children with VI, this study aims to scientifically modify and validate ADOS-2^®^, which is a ‘gold standard’ autism diagnostic observation assessment method (Gotham et al., 2007). This will be investigated in children with congenital disorders of the peripheral visual system (globe, retina and anterior optic nerve), whose VI ranges from moderate/severe to profound reduction of vision. Although a significant proportion may have developed moderate range VI (logMAR 0.7-1.0) by school age, they are likely to have been in the severe-profound VI range during infancy and only gradually developed more functional vision (Salt et al., 2020).

A similar clinical need in deaf children (HI) has recently led to the first scientifically developed adaptation and successful validation of the ADOS-2 with this population (Phillips et al., 2022). After an initial exploratory stage of trialling different autism observation techniques and tasks, it was decided to advance primarily with the ADOS-2^®^ materials and methods with licensed agreement from the original author and publishers (Lord et al., 2012). The algorithm method used here was drawn from the revised validation study of ADOS-2 by Professor Cathy Lord and colleagues (Gotham et al., 2007).

This study focuses on children who can be assessed on a modified version of ADOS-2^®^ (Module 3). The reasons for this focus on Module 3 are:

1. Early general developmental delay in most children with severe VI prevents reliably differentiating ASD from the VI-related developmental trajectories until children are at the ‘verbally fluent’ stage,
2. Early maladaptive behaviours with ASD traits may disappear as speech and cognition improve and therefore it is recommended to wait until late preschool or early school years to see if there are continuing concerns,
3. Many children with congenital disorders of the peripheral visual system make cognitive and language progress across the early years, though there may be a significant risk of ASD irrespective of cognitive level,
4. ADOS-2^®^ Modules 1 (no consistent phrase speech) and 2 (phrase speech only) are reliant on vision for many task materials, administration and coding rating. They are difficult to modify and use reliably for children with VI.

The primary objective of this study was, therefore, to design modifications of the standard ADOS-2^®^ (Module 3) and investigate its scientific validity in use with children with VI. This would be investigated according to (1) content and construct validity, (2) discriminant criterion validity against expert clinician formulation, and (3) concurrent criterion validity against parent-rated validated social communication and pragmatic communication questionnaires. To minimise within-population heterogeneity and possible internal participant bias, the sampling would be restricted to children with congenital disorders of the peripheral visual system who are anticipated to have lower levels of co-morbid neurological complexity including intellectual disability (particularly those of the ‘simple’ type, with no known neurological involvement in the paediatric diagnosis). This contrasts with children with VI of cerebral origin where brain changes may be diffuse and widespread (Sonksen and Dale, 2002). Further, higher functioning children within the population who were ‘verbally fluent’ were selected so that the single module (ADOS-2^®^ Module 3) could be modified and trialled systematically.

The secondary objective was to take the opportunity of including in the total sample all the children of a follow-up nationally representative infancy cohort (OPTIMUM) who were now within the appropriate age range. The combined national cohort and cross-sectional sample would permit learning about the frequency and distribution of ASD as a co-morbidity in children with VI aged 4 to 7 years. If any children in this cohort were not in the ‘verbally fluent’ range they would still be included in the secondary analysis.

## Methods

A prospective cross-sectional observational design (to Standards for Reporting Diagnostic accuracy studies, STARD 2015) was used. Although a similar study has not been undertaken previously, a target sample of *n* = 100 children was estimated to be powered sufficiently for the study design. For the expected meta-analysis prevalence rate of 19% with ASD in heterogeneous populations of VI children, (Do et al., 2017), a sample size of *n* = 98 for the margin of error or absolute precision of ± 8% was considered appropriate for estimating the prevalence with 95% confidence and a potential loss/attrition of 5%. With this sample size, the anticipated 95% CI was (11%, 27%). This sample size is calculated using the Scalex SP calculator (Naing et al., 2022).

Health ethical approval and guidance of IRAS (Health Ethics committee ref. 16/LO/0378) was obtained. Original recruitment to the (OPTIMUM) cohort subsample was undertaken with the NIHR portfolio (NIHR CRN Portfolio Study ID 12367, CSP 55126). A multidisciplinary patient and public involvement committee including clinical and educational advisors, user charitable organisations and parents was set up to advise on design, execution and interpretation of the study. 

### Inclusion Criteria

Inclusion criteria for the primary objective of the study were (1) congenital disorders of the peripheral visual system (i.e. globe, retina and anterior optic nerve), (2) significant VI (moderate VI to total blindness ranges, worse than logMAR 0.7 which is the lower end of the moderate range), (3) aged 4 to 7 years, and (4) ‘verbally fluent’ in English. This included children with ‘simple’ or ‘complex’ (with specified brain involvement in the paediatric diagnosis) congenital disorders according to pre-defined taxonomy (Sonksen & Dale, 2002). Exclusion criteria included children with cerebral visual impairment, hearing, motor or neurological impairments, lack of ‘verbal fluency’ in the child, or parents with insufficient English skills for oral interview or responding to questionnaire. For the secondary objective of the study, all of the above inclusion criteria and additionally *all children* from the follow-up OPTIMUM cohort who may not yet be ‘verbally fluent’.

### Recruitment and Sampling

Recruitment was undertaken through the primary research hospital site (tertiary paediatric, Great Ormond Street Hospital London UK), a second research site for recruitment (tertiary paediatric eye, Moorfields Eye Hospital London UK), a representative national longitudinal cohort (OPTIMUM) from a previous study undertaken by the research team (Dale et al., 2017), and a national open enrolment strategy (via clinicians, specialist teachers for the visually impaired, national user organisations and newsletter publicity to parents). Any child fulfilling the inclusion criteria for the primary objective of the study and *all children* in the OPTIMUM cohort who had participated in the last time period (T3, around 3 years of age) of the previous study and could still be located were contacted and invited to participate. Referring professionals and parents were informed that the study was about investigating methods for assessing social development, social interaction, play and behaviour in young children with VI and learning more about their development. The consultant paediatrician investigator in the project team checked the vision paediatric diagnoses for inclusion purposes.

## Measures

### Modification of ADOS-2^®^

The authors designed and trialled a modified ADOS-2^®^ (Lord et al., 2012) – Module 3 (‘Verbally fluent’, Children and Adolescents) for use with children with VI aged 4 to 7 years. The test manual stipulates that ‘verbally fluent’ speech is defined as using a range of sentence types and grammatical forms, including the use of conjunctions such as “but” or “though,” roughly approximate to the language level of a typical 4-year-old. Permission was provided by Professor Cathy Lord and Westen Psychological Services for undertaking these modifications and trialling their usage in this scientific validation study. The tasks retained included those that did not require vision and had miniature objects that were recognisable through touch. < See Supplement. Figure 1 > All tasks involving picture material (cartoons, books, picture cards) were excluded. The construction task instruction was to ‘build a tower’ as well as to ‘make a pattern’ if appropriate. There were some replacements of miniature objects in the joint interactive play task to ease identification. Module 2 ‘pretend play family and furniture set’ was added as a useful play ‘press’ for the young children with VI, including addition of a novel recounted story stem to provide more opportunities for socio-emotional understanding and empathy when the examiner joined the reciprocal pretend play. A pre-recorded oral story (devised by the investigators) including an imaginary story situation, with opportunity for emotional responses and fantasy/reality paradox and joint imaginative conversation, was used as a ‘press’. Toys for the ‘break’ were chosen for suitability for children with VI such as a radio, sound-making toy, pin pattern toy and bright coloured pens for drawing if the child had sufficient vision. The wording of some of the emotions and relationship-related questions had minor script changes and omissions (for example, of adult romantic relationships) to aid suitability and comprehension of the younger child. The creating a story task was excluded in case the young child became confused by or fixed on literal identification of the items (e.g. string, playing card, paper umbrella).

For administration purposes, the toy items were introduced through multisensory means by the examiner permitting visual (where the child might look at or peer at the toys if sufficient vision available), haptic (taking the child’s hand to feel the object location and contours for tactile identification) and auditory sensory access (sound cues to aid localisation or oral language from the examiner to support symbolic representation and object labelling). The examiner provided linguistic self-description and tactile or auditory action cues to show the child what the examiner was doing with their actions with play objects when joining play. The child’s hand was taken to show the location and contours of the imaginary object locations, e.g. the hot and cold taps and the basin, of the Demonstration task.

For rating after the assessment, the examiner used the scoring ratings and descriptions in the administration manual and protocol booklet (ADOS-2^®^, Module 3 Verbally fluent Child/Adolescent, Lord et al., 2012). Three items that are highly vision-dependent were excluded (B1 Unusual Eye Contact, B2 Facial Expression Directed to Examiner, and B3 Language Production and Linked Nonverbal Communication). The scores were coded according to the existing ordinal scale of 0 (no apparent difficulty) and the values of 1, 2 and 3 (progressively greater difficulty). One item, A9 Descriptive, Conventional, Instrumental or Informational Gestures, was debated whether to include in the rating, but it was decided to retain as children would need to use descriptive gestures at least in the Demonstration Task. Coding categories were grouped according to A- Language and Communication and B- Reciprocal Social Interaction (combined A and B as Social Affect (SA) and C- Imagination and D- Stereotyped Behaviours and Restricted Interests according to the organisation of the original ADOS-2^®^ manual (Lord et al., 2012). Terminology for final algorithm groupings was SA and Restricted and Repetitive Behaviour (RRB) as in Gotham et al. (2007) and the ADOS-2^®^ manual (Lord et al., 2012).

Full details of the Protocol modifications and administration methods developed by the investigators for this study are stored with the test publishers (WPS); permission can be requested from WPS for research access purposes only.

### Cognition

Each child was tested for verbal cognition (Wechsler Preschool and Primary Scale of Intelligence, Fourth edition, WPPSI-IV 2012) using the verbal subtests of Information and Vocabulary, with real-life objects replacing the pictorial items at the beginning of the Vocabulary test. Items relying on vision to comprehend or disability-sensitive were given modified wording or omitted (e.g. ‘what colour is grass?’(omitted), ‘how many eyes do you have?’ (modified to ‘how many ears do you have?’) and rated pro-rata (treated as missing data and no score assigned or given a positive score if achieved with equivalent wording). The Similarities subtest was omitted as it is not administered with the younger children and the initial pictorial items could not be converted to real-life objects. Summed raw scores were converted to the manualised standard test scores according to the child’s chronological age, but the standardisation has not been validated on children with VI. As the two subtests do not form the standardised composite index they were used separately for analysis purposes; the Vocabulary subtest was used in the analyses reported in this paper. The children were tested for auditory working memory on the British Ability Scales (BAS-II) (Elliot et al., 1996) Digit Forward subscale, leading to T scores and percentiles. The small group from the OPTIMUM cohort who were lower functioning were assessed on the Reynell Zinkin Scale for pre-schoolers with visual impairment (Reynell & Zinkin, 1975) using methods to convert to developmental quotients (Dale & Sonksen, 2002) for descriptive purposes.

### Functional Vision

Children were assessed for detection vision using the Near Detection Scale (NDS) at standard near distance (see methods in Salt et al., 2020). They were classified according to detection vision as ‘profound VI’ (PVI) – light perception at best (NDS categories 0–1) or ‘severe-moderate VI’ (SVI/MVI) – non-light-reflecting form vision of differing size of visual lure detected (NDS categories 2–9). Recognition acuity was obtained in those within the SVI/MVI range who had sufficient vision to see the Sonksen logMAR test administered at 3 m distance initially (Salt et al., 2007; Sonksen et al., 2008) and used to classify the children according to severe, moderate and mild VI ranges (ICD-10 classification). For an interval scale to include the entire sample, children who had no vision or vision too low to be measured on the logMAR test were placed in the lowest scale point as vision worse than logMAR 2.5. Vision category levels were also classified by logMAR and detection vision estimates, according to ICD-10 classification.

### Training in Methods

The postgraduate psychology examiners were trained to reliability standards in cognitive and functional vision testing and modified use of the ADOS-2^®^ and related tasks by the experienced consultant clinician researchers (clinical paediatric neuropsychology - ND and neurodisability paediatrics - AS) and senior postdoctoral psychology research associate (ES) leading the project team. The two clinicians (ND, AS) were trained by a national trainer and experienced in administering standard ADOS-2^®^ for clinical diagnostics and had high expertise and experience in assessment methods with children with VI and ASD, including pilot use of the modified ADOS-2^®^ in the clinic context. Three videos that were not included in the final sample were used for practice purposes, with additional written guidance provided for standardisation and reliability in administration and coding. A national ADOS-2 trainer who also had experience with children with VI but was not part of the investigating team assisted in consensus ratings of the practice videos to ensure reliability in coding and to resolve any areas of uncertainty.

### Assessment of Child

The child was invited to the hospital research site for a two-hour assessment session with one break for snacks and rests. A minority were tested at home because of geographical distance from the hospital site. On arrival and after an initial warm-up, the child was tested by a first examiner for cognitive level and functional vision. The child was assessed separately and on most occasions without the parent present in the room. The child was then brought into a playful situation with a second examiner and participated in the videoed play-based assessment using the modified ADOS-2^®^ framework to elicit social interaction and communication, play and behaviour using the appropriate tasks or ‘presses’ (contexts to facilitate interaction, play and communication). A digital SONY camcorder and an external directional microphone were placed half a metre away from the child. The first examiner sat in the room and observed and kept records. The second examiner used the written guidance script flexibly with each child to ensure consistency of administration. Both examiners undertook final coding and ratings and a consensus discussion was undertaken to reach final ratings which were entered into the database. For the secondary objective of the study, the children from the OPTIMUM cohort (who were at the level of ‘no consistent phrase speech’ or ‘phrase speech only’ and lower functioning on the Reynell Zinkin Scales) participated in a videoed play-based assessment using social interactional and communication items that were trialled in the (OPTIMUM) cohort study (Dale et al., 2019).

At a similar time to the child testing session, parents sat in a separate room to fill in parent-rated questionnaires on their child’s everyday social, communication and other behaviour. This included the validated standard Social Responsiveness Scale-Second Edition (SRS-2) (Constantino et al., 2003), Vineland Adaptive Behaviour Scales second edition (VABS-II) (Sparrow, 2005), and Children’s Communication Checklist-Second Edition (CCC-2) (Bishop, 1998). The SRS-2 has not been validated with children with VI and two items were pro-rated by exclusion and treated as missing data and no score assigned: Question no. 16, ‘Avoids eye contact or has unusual eye contact’, and Question no. 65 ‘Stares or gazes off into space’. For the CCC-2: one item Question no. 14. ‘Does not look at the person s/he is talking to’ was pro-rated similarly. The VABS-II proved difficult to score with unreliable responding and missing data and the results are not included in this paper.

The semi-structured validated parent interview of the Development and Well-Being Assessment (DAWBA) (Goodman et al., 2000) was undertaken online or on the phone as an interview by the examiner where online was not feasible for the parent. It was administered prior to the assessment and when on the phone by an examiner separate from the direct ADOS-2^®^ examiner. Only sections of the DAWBA relevant to social communication difficulties were administered; these included the Strengths and Difficulties Questionnaire, Social Aptitude Scale, Friendships section, Development section and Section F, Compulsions and Obsessions. The one item related to ‘eye contact’ was not included in the interview. The parent was administered the initial ‘entry’ questions for each section (other than the Development section); if the child was not reported as having any difficulty they proceeded to the next section. If a concern was reported then the more detailed questioning in the section was undertaken. The pre-scored DAWBA results as reported verbatim were read by the paediatric clinician investigator who also viewed the videos separately after the assessment.

Across the period of the study two examiners were trained to administer and to code the functional vision and modified ADOS-2^®^ assessments and three other examiners were trained to undertake the cognitive assessment and modified ADOS-2^®^ coding for reliability purposes.

### Clinical Formulation Method

The paediatric clinician investigator viewed the video play-based assessments and the parent-reported responses on the DAWBA to reach a clinician formulation (based on DSM-5 ASD, 299.00, F84.0). Appendix S.1 provides details of the DSM-5 ASD criteria as adapted for children with VI by the paediatric clinician investigator and used previously in their clinic context. The clinician investigator reached a formulation using the combination of information from the parent (DAWBA interview) for rating Criteria A, B, C and D (DSM-5) and observation of the play-based assessment for rating Criteria A and B. This led to a three-category formulation of Category 1: *Non-spectrum*, defined as no significant social or behavioural difficulties evident, Category 2: *Borderline-ASD*, defined as below full threshold but reaching partial criteria for ASD threshold, and Category 3: *ASD*, defined as reaching full threshold for DSM-5 classification for ASD including criteria for Social Communication/Social Affect (SA) and Restricted and Repetitive behaviours (RRB).

### Masking

The paediatric clinician investigator was ‘masked’ from and not informed of the modified ADOS-2^®^ examiners' scored ratings and in reverse each examiner was ‘masked’ from the DAWBA descriptions and the clinician investigator’s formulations; none of the children were known to the clinician investigator in the clinic context.

### Interrater Reliability

Intra-class correlation coefficients (ICC) were calculated from testing the inter-rater reliability of twenty videos undertaken by one examiner team and compared with the ratings of the other examiner team who observed the videos a number of months later and had not been involved with the child or parent.

### Statistical Analysis

Data was collated and analysed on the IBM SPSS Statistics 25.0 database. For correlational or comparative statistical analyses, all distributions were examined for parametrical or non-parametrical and the statistics selected accordingly. For frequency estimates, the percentages of ASD categories (‘borderline-ASD’ and ‘ASD’) were calculated according to (1) clinician formulations, and (2) the new diagnostic thresholds of ‘High Risk for ASD’ and ‘Low Risk for ASD’ arising from validation of the modified ADOS-2^®^ (Module 3) analyses. These were also examined according to vision level categories.

For effect estimates, confidence intervals were provided for the correlations. Effect sizes were calculated through Cohen’s d (parametric) or partial eta squared (non-parametric). The significance level was set at alpha < or = 0.05. A principal components analysis with rotation method (oblimin with kaiser normalisation) to extract main components (rotation with five iterations) and requesting two components was undertaken to establish whether the individual children’s scores on all items fell in line with the DSM-5 two factor classification of ASD. The KMO (Kaiser-Meyer-Olkin) measure would measure the proportion of variance among the variables that can be derived from the common variance.

The individual item results were analysed using similar terminology and the validation and algorithm method of ADOS-2^®^ of Gotham et al., 2007. Following this, the ‘preferred’ items for the ‘non-spectrum’ group (clinician investigator formulation) were those where no more than 20% of this domain group scored 2 or 3 on the particular item and the ‘ASD’ group (clinician formulation) were those where no more than 20% of the domain group scored 0 on the particular item. Items were thus classified according to whether they were ‘preferred’ items for the ‘non-spectrum’ or ‘ASD’ domain categories with those items summed together on the ‘ASD’ domain category forming the algorithm for validation testing. A principal components analysis requesting two components was undertaken to establish whether the individual children’s scores on the selected algorithm items fell into the original categories of Social Affect (SA) and Restricted Repetitive Behaviours (RRB) according to Gotham et al., 2007 and in line with DSM-5 classification of ASD.

A receiver operating curve (ROC) analysis was undertaken to establish whether the total sum of the selected algorithm items (for the total scores and separately for those algorithm items that fell into the original categories of SA and RRB) had sensitivity and specificity for classifying each child according to the ‘non-spectrum’ or ‘ASD’ categories from the clinician investigator formulation. Area under the Curve (AUC) was calculated and Positive Predictive Values (PPV). Cut-off thresholds of ‘Low Risk for ASD’ and ‘High Risk for ASD’ were established by the highest Youden Index (J). The parental reported questionnaire results (SRS-2, CCC-2) were grouped into those above and below the cut-off thresholds (‘Low Risk for ASD’ and ‘High Risk for ASD’) and scores on the Total and individual subscales were compared using the Mann-Whitney test.

## Results

The design and methodology of the study (known as the DAiSY project) led to the primary and secondary objectives of the study being successfully met according to the original research protocol.

### Participation and Descriptives

One hundred and twenty-nine children (129% of target sample size) were ascertained over a period of 24 months, originally aimed for 18 months but with additional six-month funded extension, with 29 (22%) not participating. One hundred (78% of those ascertained) consented and participated in the study; this was viewed as a satisfactory ascertainment/participation ratio of 1:0.77. Fifty-four of the sample (54%) were followed up from the previous OPTIMUM cohort project which was completed at the time of this study (Dale et al., 2019). See Appendix S.2 Fig. 1 for flow diagram of ascertainment, participation and non-participation and missing data. Sixty-one (61%) were identified through the national health service (46, 46% of total sample at the primary hospital site), 31 (31%) from the education service (mainly specialist teachers for the visually impaired) and 8 (8%) from self-referral (6, 6%) or parent charities/voluntary sector. The child ages were mean of 5 years, 6 months, SD 10.44 months, range 4 years, 1 month-7 years, 10 months. Fifty-nine (59%) were male. Ninety-five (95%) were full or near full-term and 5 (5%) were preterm (27–34 weeks’ gestation). Seventy-nine (79%) mothers provided demographic education information (21, 21% missing data); 13 (16%) education in primary and secondary school but not higher A-level certification, 17 (22%) higher A-level/ upper school leaving certification, 49 (62%) higher education including undergraduate or postgraduate degrees. Of the 80 (80%) mothers (20, 20% missing data) providing information about ethnicity, 55 (69%) were White/White British, 10 (12%) Asian/Asian British, 7 (9%) Black/African/Caribbean/Black British and 8 (10%) mixed, multiple or other.

Of the visual measurements (97, 97%, 3 missing data), 49 (51%) were in the moderate (7 in the mild range), 28 (29%) severe and 20 (21%) in the profound VI range according to Sonksen & Dale (2002) with profound VI being light perception at best. See < Supplement. Table 1 > for frequency of visual level categories. Thirty children (31%, *n* = 97) were in the ICD-10 classification for ‘blindness’ (categories 4, 5 and 6) and had near detection estimates or measured acuity worse than 3/60 or logMAR 1.3. An additional 18 (18.5%) children were in the severe visual impairment range (vision worse than 6/60 or logMAR 1.0, equal to or better than logMAR 1.3). Of those with a recognition visual acuity measure in the whole sample (81, 81%, 4 missing data), the mean was logMAR 0.98 (SD 0.38) which is on the border of the severe VI category, range 0.23–2.22. In the ‘verbally fluent’ sample analysed (n = 83, 3 missing data), 37 (46%) were in the severe-profound VI range and the others mainly in the moderate VI range (see Supplement. Table 1).

The children’s visual disorders and distribution between profound and severe/moderate VI are listed in Supplement. Table 1. In relation to vision diagnoses, 81 (81%) had ‘simple’ and 15 (15%) had ‘complex’ congenital disorders of the peripheral visual system (*n* = 4 missing data). Of these, 14 in the ‘complex’ range had septo-optic dysplasia.

Ninety children undertook a cognitive assessment with the WPPSI-IV. Combining the two verbal subtests into the standardised index for descriptive purposes, 90 (90%) of the total sample were in the average range for their age norms with a mean of 97.0, SD 15.0, with a mean scaled score of 9.42 (SD 3.13, range 1–18) on the Information subtest and a mean scaled score of 10.43 (SD 3.24, range 3–18) on the Vocabulary subtest. The children were also tested for working memory (BAS-II) and achieved a mean T score of 0.50 (SD 0.13, range 0–72); the mean percentile was 52.99 (SD 31.48) which is average range.

For the primary objective of the study, the play-based assessment using the modified ADOS-2^®^ (Module 3) was undertaken with the sample who were ‘verbally fluent’ (*n* = 83 children, 83%, 3 missing data). For the secondary objective, an additional group of 14 (14% of the total sample) children from the OPTIMUM cohort were included. Of these, six (6%) were ‘phrase speech only’ and eight (8%) were ‘no consistent phrase speech/preverbal’ levels. Ten were cognitively lower functioning and were assessed with the Reynell-Zinkin Scales. Eight (9%) of the ‘simple’ visual disorders (*n* = 85, 4 missing data) and seven (47%) of the ‘complex’ visual disorders (*n* = 15) were below the ‘verbally fluent’ level. Of the 15 children in the profound VI range, 6 (40%) had ‘phrase speech’ or ‘no consistent phrase speech’.

### Frequency of ASD Categories (Clinician Formulation)

See < Supplement. Table 2 > for frequency of ‘non-spectrum’ or ‘ASD’ categories according to clinician investigator formulation. The proportion rates of ASD were 10.8%; 32.5% (‘ASD’; ‘borderline-ASD plus ASD’) in the ‘verbally fluent’ sample. The proportion rates of ASD were 20.6%; 39.8% (‘ASD’; ‘borderline-ASD plus ASD’) in the total sample including the additional children who were rated as ‘no consistent phrase speech’ / ‘phrase speech only’. In the latter group of children, the rates of ‘ASD’ and 'borderline-ASD plus ASD’ were 78.6%; 85.7%.

#### Vision Level

The proportions of ‘ASD’ categories were significantly associated with vision level in the ‘verbally fluent’ sample (*n* = 83, 3 missing data), with greater proportions of ‘ASD’ categories with greater severity of vision reduction (Pearson Chi square = 15.5, df = 4, *p* = 0.004). < See Supplement. Table 3 > Proportions of ‘ASD’; ‘borderline-ASD plus ASD’ were 7%; 21% in moderate VI, 10%; 31% in severe VI, and 33; 89% in profound VI categories respectively.

### Validation of Modified ADOS-2^®^ (Module 3)

The following sections present the results of the primary objective of the study.

### Content Validity

The modified assessment methods and ‘presses’ worked sufficiently well to permit ratings on all included items of the full construct of Social Affect (SA), Imagination/Creativity (C) and Restricted Repetitive Behaviours (RRB) of the modified ADOS-2^®^ Module 3 recording form. In relation to inter-rater reliability, ICC with two-way random effects, single measures and absolute agreement showed strong positive significant ICC for SA (ICC 0.93, p < 0.001) and RRB (ICC 0.98, p 0.<001) and Total (ICC 0.95, p 0.001) items (SA, RRB and C) of the modified recording form.

### Construct Validity

Following rating of the videos, the sample had a raw score of M = 8.45 (SD = 6.56; range 0–29) on the items in the SA subscale, a raw score of M 1.57 (SD 1.98; range 0–8) on the items in the RRB subscale, and a raw score of M 10.52 (SD 17.70; range 0–39) on the Total scale (SA, RRB and C). Internal consistency of the items was high for the SA items (Cronbach α 0.90) and acceptable for RRB items (Cronbach α 0.62). Scores on SA had a strong significant correlation with the Total scores (Spearman rho 0.939, *p* < 0.001) but a weak non-significant correlation with RRB (rho 0.212, p 0.055).

#### Age, Gender and Cognitive Ability and Vision Level

The correlations between age and SA and Total scales (ρ -0.35, p 0.001; ρ -0.26, p 0.05) were significant weak inverse respectively, and non-significant for RRB (ρ 0.12, p 0.30). There were no gender differences in the SA category between males (M 8.50; SD 6.90) and females (M 8.37; SD 6.17) (t(81) 0.09, p 0.93 (95% CI -2.79-3.05) Cohen’s d 0.02); in the RRB category between males (M 1.88; SD 2.09) and females (M 1.14; SD 1.75), (t(81) 1.68, p 0.10 (95% CI -0.13-1.60) Cohen’s d 0.38), and in the Total category between males (M 11.00; SD 8.45) and females (M 9.86; SD 6.61, (t(81) 0.67, p 0.51 (95% CI -2.28-4.56) Cohen’s d 0.15). The correlations between Verbal cognition (Vocabulary) and SA and Total scales (ρ -0.44, p 0.001; ρ -0.39, p 0.001) were significant weak inverse respectively, and non-significant for RRB (ρ 0.01, p 0.90). No significant difference in frequencies across the categories was found between the ‘simple’ (*n* = 71) and ‘complex’ (*n* = 9) vision disorder categories: (Mann-Whitney U 241.5 p 0.23; 229.0 p 0.15; 265.00 p 0.41 for SA, RRB and Total raw scores respectively).

In relation to the logMAR acuity scale (including those untestable coded as logMAR worse than 2.5), SA scores had minimal non-significant correlation with vision level (rho 0.161, p 0.154). The RRB scores had a moderate significant correlation with vision levels (rho 0.513, *p* < 0.001). A weak significant correlation was found between Total scores and vision level (rho 0.323, p 0.003). Examining those children who had sufficient vision to be tested on logMAR test (*n* = 72, 3 missing data), the correlations between visual acuity (logMAR) and SA and Total were non-significant respectively (ρ 0.10, p 0.43; ρ 0.18, p 0.13) and significant weak positive for RRB (ρ 0.34, p 0.01).

### ASD Domain Analysis and Algorithm

Using principal components analysis, a first analysis extracted four components explaining 61.9% of variance (KMO 0.82). A further analysis extracting two components < see Supplement. Table 4 > explained 48.1% of variance (KMO 0.82). The two components corresponded to mainly 1) items A and B (SA), with C - Imagination/Creativity aligned with SA, and 2) items D mainly aligned with RRB.

According to the ‘preferred item’ pooling and algorithm computation, 12 items reached threshold criterion for the ‘ASD’ domain category (clinician formulation). Eight items were in the SA scale – including 6 that were borderline reaching slightly over the threshold criterion (range 21-25%) and considered of clinical relevance and therefore included in the final algorithm. One item was Imagination/Creativity and three items were in the RRB scale (with 2 items reaching well over the threshold criterion: D4 Excessive interest in or reference to unusual or highly specific topics or objects or repetitive behaviours, and A4 Stereotyped/ idiosyncratic use of words or phrases but included for clinical relevance). < See Supplement. Table 5>.

These twelve items were subjected to principal components analysis (extracting two factors). < See Supplement. Table 6>. The items fell into the same components and were interpreted as SA and RRB as in the original algorithm (Gotham et al., 2007) with the addition of Creativity/Imagination that also fell in the SA factor. The KMO measure indicated good sampling adequacy (KMO 0.75) and the Bartlett’s test of sphericity was significant (χ^2^(66)359.3, *p* < 0.001). The requested 2 component extraction with oblimin rotation did not eliminate any of the 12 items. The communalities table showed all values > 0.3, suggesting that the items shared common variance with other items. The components of SA and RRB accounted for 36.0% and 13.8% respectively (total 49.8% of variance).

Internal consistency for these algorithm items was high for SA (9 items) (Cronbach α 0.83) and moderate for RRB (3 items) (α 0.61). Item analysis revealed that taking any items out would not have improved the scale substantially and therefore items were not deleted.

### Discriminant Criterion Validity

A ROC analysis was conducted to estimate the sensitivity and specificity of the Total algorithm (SA, RRB) scores and SA and RRB scores separately for predicting the clinician formulation ratings of ‘non-spectrum’ versus ‘ASD’ categories (0,1 codes) or ‘non-spectrum’ versus ‘borderline-ASD plus ASD’ categories. < See Supplement. Table 7 > for AUC, Youden Index J and Positive Predictive values (PPV). The algorithm scores showed strong AUC and high sensitivity and specificity to ASD domain categories (with exception of RRB algorithm to ‘borderline-ASD plus ASD’ categories where it showed moderate sensitivity). Cut-off thresholds leading to the strongest sensitivity and specificity for the algorithms was identified using the Youden Index.

The sample was divided into two groups according to the cut-off level (Youden Index) of the Total algorithm and classified as ‘Low Risk for ASD’ (< score of 7.5) and ‘High Risk for ASD’ (*≥* 9.5).

#### Vision Level

Chi-square tests revealed a significant association between differing vision level and the Total algorithm score (SA plus RRB) for ‘Low Risk for ASD’ (< 7.5) and ‘High Risk for ASD’ (> 9.5) < see Supplement. Table 8>, with greater likelihood of ‘High Risk for ASD’ at more severe levels of VI (χ^2^(2, *N* = 66) 9.60, p 0.008). A significant association was also shown between vision levels and ‘Low Risk for ASD’ (<7.5) and ‘High Risk for borderline-ASD plus ASD’ (*≥* 7.5) and vision levels (χ^2^(2, *N* = 80)12.41, p 0.002). See the proportion rates for different VI categories in Table 8.

### Concurrent Validity Against Parent-Rated Questionnaires

A Mann-Whitney test was used to compare scores in the ‘Low Risk for ASD’ and ‘High Risk for ASD’ groups on the two parent-rated questionnaires: SRS-2 and CCC-2. Eighty-one parents (n = 5 (5%) missing data) filled in the SRS-2. Forty-one (51%) were in the normal range, 17 (21%) were in the mild elevated range, 17 (21%) in the moderate range and 6 (7%) in the severe range (with 23 (28%) in the moderate-severe range on the scale). < See Supplement. Table 9 > shows significant moderate effect differences on the SRS-2 DSM Social Communication and Interaction and DSM Repetitive Interests and Behaviours and large effect differences on the Total T-scores between the Low Risk and High Risk groups.

Eighty parents (*n* = 6 (6%) missing data) filled in the CCC-2. The mean General Communication Composite was mean scaled score of 65.31 (SD = 19.58, range = 97 (24–121), mean 27th percentile). < Supplement. Table 10 > shows negligible non-significant effects on the CCC-2 scales between the ‘Low Risk for ASD’ and ‘High Risk for ASD’ groups, with exception of Social Relations and Interests Scale which showed moderate effects.

## Discussion

A cross-sectional observational investigation to scientifically design and develop an autism observational assessment methodology for children with VI was undertaken, focussing on modification and validation of ADOS-2^®^ (Module 3). This primary objective of the study was undertaken with 86 (3 missing data) children aged 4 to 7 years with ‘verbal fluent’ language level, from a total recruited sample (*n* = 100) with rare congenital disorders of the peripheral visual system and moderate to severe to profound VI. Half of the total sample were from an infancy national cohort (OPTIMUM) recruited at 8–15 months who were now at the appropriate age for follow-up in this study. A small proportion of these children had only phrase speech or no consistent phrase speech at all but were retained for the secondary objective of the study.

The ascertainment rate, national geographical recruitment strategy, rate and distribution of heterogeneous ‘simple’ and ‘complex’ congenital disorders of the peripheral visual system, maternal education, ethnic minority demographics were in line with national representative population statistics (see too OPTIMUM, Dale et al., 2017). This provided further confidence in the generalisability of the frequency estimates of ASD categories in this study, though it was not designed as a primary population prevalence study.

Around half of the ‘verbally fluent’ sample (and total sample) had vision worse than logMAR 1.0 and were in the severe to profound VI categories. They participated in the assessment peering closely at the objects if any functional vision available or using auditory attention and haptic (tactile) senses and additional auditory and haptic cues and verbal labelling and explanations of the assessor. A quarter (25%) in the ‘verbally fluent’ sample (and nearly one third (31%) of the total sample) were in the educationally ‘blind’ range with no vision, light perception or low detection vision only. These children had vision worse than logMAR 1.3 and were likely to be tactile learners in education. This corresponds with the estimates of blindness range in 1 in 4 children with congenital disorders of the peripheral visual system in the UK. Communication solely through language was a primary mode of communication for most of the children; most could not use integrated eye contact, referential gaze or gestural communication combined with their vision if available.

Most of the ‘verbally fluent’ sample (and the total sample) were in the average range on the verbal reasoning index of the WPPSI-IV Scales. Most children (including the OPTIMUM cohort) had sufficient fluent speech to undertake the modified Module 3 of ADOS-2^®^. The small proportion with ‘no consistent phrase speech’ or ‘phrase speech only’ (and likely intellectual disability by this age range) were found in roughly similar proportions from both the OPTIMUM follow up cohort and the cross-sectionally recruited sample. Their frequency rate of 14% was roughly in line with (though slightly below) previous estimates for intellectual disability in ‘simple’ congenital disorders of the peripheral visual system (Dale & Sonksen, 2002) and our previous estimate from the OPTIMUM study (Dale et al., 2019). These findings support our argument that school-aged children with ‘verbal fluent’ level are likely to be the majority population in a representative sample of ‘simple’ congenital disorders of the peripheral visual system. It is therefore important to have means of assessing reliably for ASD in this population by 4 to 7 years.

According to the clinician investigator formulations the proportion rates of ‘ASD’ were 10.8–32.5% (‘ASD’ or ‘borderline-ASD plus ASD’) in the ‘verbally fluent’ primary sample. This may be an underestimation as the clinician investigator did not have access to full relevant information about the child or to the assessment examiner ratings. This suggests that 1 in 3 children in this population, who are doing well cognitively and in expressive language ability, may still be at risk of ASD symptoms. It may be more difficult to establish their ASD risks at an earlier age because of the challenges of differentiation from the general early delays associated with significant VI (see Introduction).

A final justification as discussed in the Introduction was that ADOS-2^®^ Module 3 was potentially modifiable for this VI population. Our findings support this argument. Previous modifications to ADOS-2^®^ in small scale studies have proved feasibility but not established full psychometric validity of their modifications (Fazzi et al., 2019; Williams et al., 2014; Stevenson & Tedone, 2024). We believe that we have gone beyond these previous studies and reviews of the literature. Modification of materials and mode of administration and scoring of the ADOS-2^®^ Module 3 with the ‘verbally fluent’ sample were shown to be developmentally feasible. Content validity was found for the calibrated ratings of social communication and restricted and repetitive behaviours (SA, RRB and C - Imagination/Creativity), as in the original ADOS-2^®^ manual (Gotham et al., 2007; Lord et al., 2012) other than the excluded vision-dependent items. Although the mean Total scores were elevated, there was within-population heterogeneity and a wide distribution of scores. High inter-rater reliability in scoring of SA, RRB and Total Scores was reassuring, in view of previously reported concerns regarding difficulty in reaching reliability in scoring the child’s intentions and behaviour during a social communicative behavioural assessment (see Introduction).

The expert clinician investigator was able to use the video observations of the modified ADOS-2^®^ (Module 3) assessment and DAWBA parental reporting to categorise the children as ‘non-spectrum’, ‘borderline-ASD’ and ‘ASD’ categories according to the modified DSM-5 ASD diagnostic framework.

In relation to construct validity of the modified ADOS-2^®^ (Module 3) assessments, the calibrated raw scores on SA strongly and RRB moderately correlated with those on the Total Scores, but SA and RRB showed little correlation together. As has been demonstrated previously with typically sighted children (Gotham et al., 2007; Huss et al., 2014) and DSM-5 ASD classification, these domains appeared to distribute independently or ‘dissociate’. The study demonstrated good internal coherence for the Total and SA items and the two-component model (SA and RRB) accounted for nearly fifty per cent of variance. RRB scores were adequate for internal coherence, but not as high as SA (see too Gotham et al., 2007). It has been suggested that this difference in internal coherence of SA and RRB may be partially an observational assessment problem as SA (and ‘absence’ of social communicative behaviours) is more frequently witnessed and easily measured in the time and context-limited assessment than RRB (requiring ‘positive’ signs of abnormal behaviours such as hand flapping or behavioural compulsions that may not occur in the absence of habitual triggers) (Huss et al., 2014). The Total and SA calibrated scores (but not RRB) had significant but weak inverse effect sizes with other child developmental factors of chronological age and verbal cognition (Vocabulary) and no significant differences according to gender, suggesting that other child developmental factors were not very explanatory in the Total, SA or RRB score variance in the ‘verbally fluent’ sample.

Further construct validity was determined by use of the previous algorithm method developed by Professor Cathy Lord’s team which could distinguish whether individual items were more likely to be in the ‘non-spectrum’ or ‘ASD’ groups (Gotham et al., 2007). Ten of the items distinguished between the ‘non-spectrum’ group and ‘ASD’ group (clinician investigator formulation) and formed ‘preferred’ items for the final algorithm, showing that this method could also be utilised with the VI population. Two further items in relation to compulsions and rituals and excessive interest or reference to unusual or highly specific topics or objects or repetitive behaviours were less clear-cut in segregation but were included for clinical relevance. The algorithm was supported by strong internal consistency and coherence and the principal component analysis (requesting two components) also fell into the two-factor model (SA and RRB) accounting for fifty per cent of the variance.

Of theoretical and clinical interest is the similarity and difference of items included in the new algorithm identified here as compared to the original one with typically sighted children (Gotham et al., 2007). Although six items overlapped, six were novel and distinct and five others in the original algorithm were omitted. This finding underlines that children with VI who were at Module 3 level needed a different algorithm from the typically sighted child and it is henceforth referred to as the VI algorithm. The item distribution revealed that these higher functioning children with VI could show social motivation to engage in two-way conversation, share enjoyment in interaction and respond positively in quality and yet may still be at high risk of ASD. This may correspond more to typically sighted higher functioning girls with ASD, who have also been found to have higher social motivation than in the typical male ASD phenotype (Hull et al., 2020).

The summed scores of the VI algorithm showed strong predictive and discriminant criterion validity, with strong significant AUC 1.00 (*p* < 0.001), PPV of 100% (CI 95%) and very high sensitivity and specificity (1.00, 1.00 respectively) of the Total scores with the clinician investigator formulation categories (‘ASD’ compared with ‘non-spectrum’). Moreover high sensitivity and specificity of the SA (AUC 0.97 *p* < 0.001, 1.00, 0.82) and RRB (AUC 0.91, *p* < 0.001, 0.89, 0.91) scores were found. Similar patterns were found for the combined ‘borderline-ASD’ and ‘ASD’ category compared with ‘non-spectrum’, other than a slightly lower sensitivity (but high specificity) of the RRB scores.

Of notable empirical interest, stereotyped idiosyncratic use of words and phrases or sensory interests and hand-finger mannerisms were considered so widespread in this population that they were previously referred to as ‘blindisms’ and ‘expected VI-related behaviours’ for very young children in the clinical field and literature. These symptoms are now recognised to be more likely manifestations of ASD symptomatology if they persist in chronic form over time and it has been suggested that they potentially arise from a complex interplay of vision deprivation, shared neural anatomical pathways and behavioural development (Jure, 2022). They do however reveal the complexity of development for all young children with VI in the early years when these behaviours are commonplace. However in our study the high sensitivity and specificity of the RRB scores for the ‘ASD’ category versus ‘non-spectrum’ does suggest that they are more confined to those with a high risk of ASD, at least in those children with VI who are ‘verbally fluent’ and higher functioning.

The study results showed that a diagnostic threshold ‘cut-off’ could be established through ROC and Youden Index statistics and that this led to separating the ‘High Risk for ASD’ (and a slightly lower ‘Borderline-ASD’ group) subgroup from ‘Low Risk for ASD’ subgroup. Concurrent criterion validity was demonstrated by comparing the groups' scores on parent-rated social communication questionnaires; there were significantly higher scores showing greater social communication weaknesses on the SRS-2 questionnaire in the ‘High Risk for ASD’ group (with medium effect size) compared with the ‘Low Risk’ group. These differences were found on the SRS-2 Total T-score and DSM Social Communication and Interaction T-score and most of the subscales, with the exception of ‘social awareness’ and DSM Repetitive Interests and Behaviours T-score.

Contrastingly, the CCC-2 questionnaire results which focus on pragmatic language did not show concurrent criterion validity; negligible differences in areas like semantics, inappropriate initiation, stereotyped language and use of context were found between the ‘High Risk for ASD’ and the ‘Low Risk for ASD’ groups, except for CCC-2 Social Relations and Interests subscales. Although not reported here, the mean difficulty scores were found to be significantly higher than the manual norms for both groups suggesting that the total cohort was at risk in pragmatic language. Other studies have shown similarly in school-aged children with VI and average level-superior linguistic skills and weaker pragmatic language skills (Bathelt et al., 2017; Tadic, 2010). Deprivation of contextual visual social information may hinder development and use of expressive pragmatic language skills to support communication and interpretation of others’ communicative intentions and meaning and development of social cognition, theory of mind and perspective-taking of others (see studies of behavioural and neural studies of social cognition in children with VI; e.g. Bathelt et al., 2017; Peterson et al., 2000). Although this complex developmental process is not yet understood, it is noteworthy that the item of B5 ‘Comments on other’s Emotions/Empathy’ fitted in our VI algorithm and empathy has been viewed as part of contextual social cognition (Melloni et al., 2014).

The secondary objective of the study revealed further insights into the frequency and distribution of ASD outcomes according to level of visual acuity and degrees of impairment, including the small additional proportion of children who had ‘no consistent phrase speech’ or ‘phrase speech only’. The clinician formulation rates of 21–40% (‘ASD’; ‘borderline-ASD plus ASD’) in the total sample and 11–33% (same categories) in the ‘verbally fluent’ subsample were in line with prevalence estimates from other studies (such as the meta-analysis estimated rate of 19% (95% CI 13–25%, systematic review in Do et al., 2017). In contrast, the small subgroup of children who had ‘no consistent phrase speech’ or ‘phrase speech only’ had clinician investigator formulation rates of 79–86% for ‘ASD’ or ‘borderline-ASD plus ASD’. This suggests an extremely high co-morbidity of ASD in those who had VI and intellectual disability, though this was a small unplanned group and any conclusions are tentative. Nevertheless, it fits with the previous literature of higher rates of ASD in children with visual impairment and lower cognition (Do et al., 2017), suggesting that ‘verbally fluent’ and average or superior range verbal cognition might be playing some protective mechanism against developing ASD in children with VI (Bathelt et al., 2017).

Over half of the sample did not raise substantial concerns from the video ratings or DAWBA reports and were rated as ‘non-spectrum’ by the clinician investigator, suggesting that ASD outcomes in this vulnerable population in this age range may not be inevitable. Children may learn to socially interact and communicate and have reciprocal communication and develop symbolic play and socially related interests even in the absence of vision or very low levels of vision including insufficient vision to discern detailed social facial expression or gestural communications or other social contextual cues.

The clinician investigator formulations were strongly associated with severity of vision level in the ‘verbally fluent’ sample. This is in line with other literature showing a relationship between higher prevalence of ASD and greater degree of VI (Do et al., 2017). The rates of ‘ASD’ or ‘borderline-ASD plus ASD’ positively increased from 7 to 21% (respective categories) in the moderate vision level, to 10–31% in the severe and 33–89% (same categories) in the profound level of VI. The literature has shown the particular risk for general development in young children with very severe-profound VI (see Introduction) and this study reinforces further their higher risk for ASD, even if developing ‘verbally fluent’ speech by this age. Small graduations of improvement in vision across the logMAR scale showed no significant association with the calibrated raw scores on Total or SA (and only with RRB) scales of the modified ADOS-2^®^ (Module 3). It appears that risk of SA or Total raw scores is not necessarily reduced by possibly more detailed vision by this age period that might permit basic face processing or following some non-verbal communication cues at nearer proximity. Of interest but needing further research, the small proportion of children with ‘complex’ vision disorders (and additional known brain disorder in the paediatric diagnosis) in the ‘verbally fluent’ range showed no difference in frequency of SA, RRB or Total calibrated raw scores to those with ‘simple’ vision disorders suggesting further the protective factors of higher intelligence and language.

Across all vision levels there were individual children who fitted into the ‘High Risk’ categories according to the VI algorithm cut-off thresholds, rising from 14 to 26% (‘High Risk for ASD’ to ‘High Risk for borderline-ASD plus ASD’) in the moderate range. This rose further to 24–41% in the severe range, and 44–89% in the profound VI range. The growing evidence from this study and others (Do et al., 2017) suggests that vision deprivation and lack of access to environmental visual stimulus is a potential causal factor in relation to the aetiology of ASD. There may be other contributory causal factors too, such as genetics. Most congenital disorders of the peripheral visual system are inherited and multiple eye genes may be expressed in the eye and brain; some of these like *PAX6* (commonly found in aniridia) and *SOX2* (anophthalmia) have been implicated in brain development and ASD (Kikkawa et al., 2019; Moen et al., 2017). Infancy vision (such as for social scenes) may also be under genetic control and atypical for ASD (Constantino et al., 2017).

### Limitations

Although an a priori power calculation was undertaken on the basis of prior literature, the study was potentially weakened by having 86 (3 missing) rather than 100 children in the final ‘verbally fluent’ subsample. This is still one of the largest study groups of children with mainly ‘simple’ congenital disorders of the peripheral visual system at this age and in the higher functioning range to be reported. The highly significant results and effect sizes in the direction anticipated suggests that this slightly lower number of children is still sufficiently powered. Future studies can base their power analyses on the parameters of this new study. Investigation of a nationally representative sample of children with rare heterogeneous congenital disorders of the peripheral visual system supports confidence in generalisability of results across this population.

However there are limitations to the study methods. The clinician investigator based their formulations on viewing the same video assessments as the modified ADOS-2^®^ examiners; this may have led to a positive skew towards the calibrated rating scores of the examiners (although both were ‘masked’ from each other’s formulations and ratings). The parental responses on the DAWBA were however only available to the clinician investigator providing additional independent data. Due to resource and time constraints of the study, there was only one clinician investigator available to provide the formulations without reliability testing with another clinician. The autism observational methodology of the modified ADOS-2^®^ (Module 3) has not been validated by an independent multidisciplinary team and their diagnostic decision-making (including full vision, paediatric and developmental history of the child with and without access to the video assessments); this is planned for future feasibility testing in the clinic context. There may be a participant information bias for one of the examiners who had previously examined many of the children in the longitudinal OPTIMUM cohort between one to three years old; however the other examiners had not known this cohort and inter-rater reliability between them was high. A written script to guide the examiners enhanced standardisation and reliability and would reduce the influence of any pre-existing knowledge of the child. A further limitation is the reliance on parent-rated questionnaires (SRS-2, CCC-2) for concurrent validation as there is no other reliable validated ‘gold standard’ behavioural observational assessment tool for this population. However limiting the sample to a narrower age band and similar cognitive and language level was likely to reduce some of the recognised non-ASD factors such as age, language and cognitive level which might also influence the SRS-2 scores (Huss et al., 2013). Although the children were considered as being ‘verbally fluent’ it is likely that their language skills are not necessarily comparable to typically sighted children at this age (Mosca et al., 2015). It is difficult to assess for expressive language ability in this population but it is possible that there is some additional confound of assessment performance which needs further detailed consideration in the future.

### Future Areas of Research and Practice

Future research may build on this potentially more reliable ASD assessment methods to investigate the aetiology or causation, risk factors and mechanisms (including genetics) in the co-morbidity of VI and ASD and the longer-term individual trajectories and outcomes which is outside the scope of this study. With multiple measures undertaken from one year old in half of this sample (OPTIMUM cohort), there is opportunity for a retrospective longitudinal study to consider antecedents and early risk and protective factors, such as targeted early intervention and parenting styles, and ASD developmental outcomes and this analysis is planned.

Future research should investigate the reliability and validity of a similarly modified ADOS-2^®^ (Module 3) for the wider population of children with VI across the older lifespan, including those with brain origin cerebral VI and aged 8 years and older. It will be of interest whether the same VI algorithm is the best fit for the identification of ASD. It is our clinical experience that significant numbers of older children with VI, including in the higher functioning range, are referred to our developmental vision clinic service with queries about ASD. This paper did not report on the play-based assessments of the subsample of children who were lower functioning with ‘no consistent phrase speech’ or ‘phrase speech only’, other than the clinician investigator formulations derived from viewing the assessment videos. Research will need to continue to design and validate the appropriate observational assessment methodology for children in this lower developmental age range (e.g. Absoud et al., 2011); research analysis from the OPTIMUM cohort is mapping early social developmental norms at two and three-year-olds in this population, which are currently unknown.

These convergent results point towards a potentially valid and reliable assessment toolkit including the modified ADOS-2^®^ (Module 3) and with further supplement of parent interview and questionnaires including the SRS-2 for confidently distinguishing ASD in children with VI in this age range. Future research is needed to test this out in the clinical context. This modified assessment method including the ADOS-2^®^ (Module 3) could ‘add value’ to existing routine clinical practice by reliably identifying ASD in children with VI (Dale & Salt, 2022). It will need embedding in a comprehensive clinical assessment including information from the parent on the child’s developmental history, assessments such as verbal cognition and language, reports from other clinicians and educators and a multidisciplinary team formulation (Dale & Salt, 2022). Clinicians who are already trained and expert in ASD assessment and ADOS-2^®^ will need further specialist training including regular reliability updates.

## Summary

The study findings are promising and suggest that it is feasible to systematically differentiate social communication difficulties and ASD profiles in children with VI. The modified ADOS-2^®^ (Module 3) provides a potentially valid and reliable assessment tool, with its new scientifically derived diagnostic algorithm and threshold cut-off for identifying children at ‘High risk for ASD’ in this age range. It appears scientifically ‘fit for purpose’ for next stage clinical feasibility testing of the assessment methods. This will potentially lead to an internationally accepted diagnostic ASD assessment methodology for children with VI, in line with those who are typically sighted. This may ensure that these children have their ASD-related needs identified and receive appropriate intervention and their parents and educators have better information, guidance and support. The results of the secondary objective of this study show a significant risk for ASD in this clinical population, with greater risk with severity of VI and lower cognitive functioning.

## Electronic supplementary material

Below is the link to the electronic supplementary material.

Supplementary Material 1
