# Supplementary material for: Modification and Validation of an Autism Observational Assessment Including ADOS-2® for Use with Children with Visual Impairment
Source: J Autism Dev Disord. 2024 Sep 9;56(1):83–99. doi: 10.1007/s10803-024-06514-z (PMC12860847; doi:10.1007/s10803-024-06514-z)
Supplement: Supplementary file 1 — Supplementary Material 1 [file 10803_2024_6514_MOESM1_ESM.docx]

**Supplementary Appendix S.1**

**Criteria for clinician formulation of ASD in children with VI**

The criteria are based on DSM-5 Criteria for ASD (299.00 (F84.0)) and modified for children with VI by the clinician investigator. Level of visual difficulty was taken into account when considering criteria in italics and not considered if vision insufficient to support these skills and behaviours.

**Autism Spectrum Disorder           299.00 (F84.0)**

Diagnostic Criteria

A.      Persistent deficits in social communication and social interaction across multiple contexts, as manifested by the following, currently or by history (examples are illustrative, not exhaustive, see text):

1.       Deficits in social-emotional reciprocity, ranging, for example, from abnormal social approach and failure of normal back-and-forth conversation; to reduced sharing of interests, emotions, or affect; to failure to initiate or respond to social interactions.

2.       *Deficits in nonverbal communicative behaviors used for social interaction, ranging, for example, from poorly integrated verbal and nonverbal communication; to abnormalities in eye contact and body language or deficits in understanding and use of gestures; to a total lack of facial expressions and nonverbal communication*. *Difficulties in criteria 2 were generally assumed to relate to visual impairment and therefore not considered as contributing to diagnosis: some limited gestural communication was expected e.g. in demonstration.*

3.       Deficits in developing, maintaining, and understanding relationships, ranging, for example, from difficulties adjusting behavior to suit various social contexts; to difficulties in sharing imaginative play or in making friends; to absence of interest in peers, *where these difficulties persisted even after compensatory strategies for visual impairment were in place to foster development of social relationships.*

B.      Restricted, repetitive patterns of behavior, interests, or activities, as manifested by at least two of the following, currently or by history (examples are illustrative, not exhaustive; see text):

1.       Stereotyped or repetitive motor movements, use of objects, or speech (e.g., simple motor stereotypies, lining up toys or flipping objects, echolalia, idiosyncratic phrases).

2.       Insistence on sameness, inflexible adherence to routines, or ritualized patterns or verbal nonverbal behavior (e.g., extreme distress at small changes, difficulties with transitions, rigid thinking patterns, greeting rituals, need to take same route or eat food every day) *where this need for adherence to routine and sameness was not explicable by visual impairment*.

3.       Highly restricted, fixated interests that are abnormal in intensity or focus (e.g, strong attachment to or preoccupation with unusual objects, excessively circumscribed or perseverative interest).

4.       Hyper- or hyporeactivity to sensory input or unusual interests in sensory aspects of the environment (e.g., apparent indifference to pain/temperature, adverse response to specific sounds or textures, excessive smelling or touching of objects, visual fascination with lights or movement) *Not explicable by need for additional sensory information due to Visual impairment.*

C.      Symptoms must be present in the early developmental period (but may not become fully manifest until social demands exceed limited capacities, or may be masked by learned strategies in later life).

D.      Symptoms cause clinically significant impairment in social, occupational, or other important areas of current functioning.

E.       These disturbances are not better explained by intellectual disability (intellectual developmental disorder) or global developmental delay

**Borderline / High Risk / or symptoms of social communication difficulties**

**Criteria**

Some deficits are seen in social communication and/or social interaction but NOT seen in both areas of Criteria A: social-emotional reciprocity and in developing, maintaining, and understanding relationships. *(Criteria 2 was assumed to be limited in children with visual impairment and not taken into account in diagnostic formulation)*

OR

Deficits are seen in Criteria A but symptoms in only 1 area of Criteria B (Restricted, repetitive patterns of behavior, interests, or activities) OR no symptoms in Criteria B

OR

At least 2 Symptoms in Criteria B but no symptoms in Criteria A

**Appendix S.2**

Figure 1 Flow diagram of ascertainment, participation and reasons for non-participation

**Appendix S.2 Table 1 Ophthalmological disorders and vision levels of the total recruited sample and comparison with national epidemiological data**

|  | Visual disorder (grouped according to primary anatomical site affected) | N=100 (%) | PVI  (n=15) | SVI  (n=85) | UK national epidemiological data ^15^  N=439 (%) |
| --- | --- | --- | --- | --- | --- |
| **1** | *Whole globe and anterior segment* | **13 (13%)** |  |  | **29  (7%)** |
| 1.1 | Microphthalmia/anophthalmia | 4 | **2** | **2** |  |
| 1.1.1 | *- additional coloboma* | 1 |  | 1 |  |
| 1.2 | Anterior segment dysgenesis | 3 |  | 3 |  |
| 1.3 | Coloboma-multiple sites | 3 |  | 3 |  |
| 1.4 | Other *- High myopia* | 1 |  | 1 |  |
| 1.4.6 | - Persistent hyperplastic primary vitreous | 1 |  | 1 |  |
| **2** | *Glaucoma (primary and secondary)* |  |  |  | **13  (3%)** |
| **3** | *Cornea (sclerocornea and corneal opacities)* | **1** |  | **1** | **7 (2%)** |
| **4** | *Lens (cataract or aphakia)* | **5** |  | **5** | **21 (5%)** |
| **5** | *Uvea* | **8** |  |  | **12 (3%)** |
| 5.1 | *Aniridia* | 8 |  | **8** |  |
| **6** | ***Retina*** | **47 (47%)** |  |  | **126  (29%)** |
| 6.2 | Retinal and macular dystrophies |  |  |  |  |
| 6.2.1 | - Cone | 6 |  | 6 |  |
| 6.2.2 | - Cone-rod | 4 |  | 4 |  |
| 6.2.4 | - Early onset retinal dystrophy   (Leber’s congenital amaurosis) | 9 | 5 | 4 |  |
| 6.2.7 | - Congenital stationary night blindness | 1 |  | 1 |  |
| 6.2.9 | - Unspecified macular dystrophy | 1 |  | 1 |  |
| 6.3 | Ocular-cutaneous albinism | 11 |  | 11 |  |
| 6.6 | Retinoblastoma | 4 | 1 | 3 |  |
| 6.7 | Other |  |  |  |  |
| 6.7.2 | - Dysplasia (inc retinal folds and Norrie disease) | 1 | 1 |  |  |
| 6.7.7 | - Ocular albinism | 7 |  | 7 |  |
| 6.7.8 | - Familial exudative vitreoretinopathy (FEVR) | 3 |  | 3 |  |
| **7** | ***Optic nerve*** | **17 (17%)** |  |  | **123  (28%)** |
| 7.1 | Hypoplasia |  |  |  |  |
| 7.1.1 | - Isolated | 10 | 2 | 8 |  |
| 7.1.2 | - Septo-optic dysplasia | 7 | 3 | 4 |  |
| 7.2 | Atrophy |  |  |  |  |
| 7.2.1 | - Primary |  |  |  |  |
| **9** | ***Other*** | **5** |  |  | **8 (2%)** |
| 9.1 | Idiopathic nystagmus | 5 |  | **5** |  |
|  | Unknown | **4** |  |  |  |
|  |  |  |  |  |  |

**MAIN TABLES AND FIGURES**

Figure caption sheet

Figure 1 Tasks or ‘presses’ in the modified ADOS-2® (Module 3, Verbally Fluent, Children and Adolescents) for children with VI

Figure 1 Tasks or ‘presses’ in the modified ADOS-2® (Module 3, Verbally Fluent, Children and Adolescents) for children with VI

| Included | Excluded |
| --- | --- |
| Construction task | Description of a picture |
| Make-believe play | Telling a story from a book |
| Joint interactive play | Cartoons |
| Demonstration task | Creating a story |
| Conversation and reporting |  |
| Emotions |  |
| Social difficulties and annoyance |  |
| Break |  |
| Friends and relationships |  |
| Loneliness |  |
| Addition  Auditory-related story |  |

Table 1 Frequency of children at different vision levels (ICD-10 classification categories) in total sample and ‘verbally fluent’ subsample

| Vision level category | Worse than | Equal to or better than | Total recruited sample (N=97, 3 missing data)  n | ‘Verbally fluent’ subsample, N=83, 3 missing data) n | Vision function categories  (Sonksen and Dale, 2022) | ‘Verbally fluent’  subsample  N=83, 3 missing data) n |
| --- | --- | --- | --- | --- | --- | --- |
|  |  |  |  |  |  |  |
| 1 Mild visual impairment | 6/9  logMAR 0.2 | 6/18  logMAR 0.5 | 7 | 6 |  |  |
| 2. Moderate visual impairment | 6/18  logMAR 0.5 | 6/60  logMAR 1.0 | 42 | 37 | Moderate VI (category 1 and 2) | 43 |
| 3. Severe visual impairment | 6/60  logMAR 1.0 | 3/60  logMAR 1.3 | 18 | 16 | Severe VI  (category 3 and 4) | 25 |
| 4. Blindness | 3/60  logMAR 1.3 | 1/60  logMAR 1.8 | 10 | 9 |  |  |
| 5. Blindness | 1/60  logMAR 1.8 | Light perception | 8 | 3 | Profound VI  (category 5 and 6) | 12 |
| 6. Blindness | No light perception |  | 12 | 9 |  |  |

Table 2 Frequency of clinician formulations of ASD according to total sample, ‘verbally fluent’ subsample and ‘no consistent phrase speech/ phrase speech only’ subsample (n, %)

| Clinician formulations | Total sample (n=98, 2 missing data) | ‘Verbally fluent’ subsample (n=83, 3 missing data) | ‘No consistent phrase speech/ phrase speech only’ subsample (n=14) |
| --- | --- | --- | --- |
| Non-spectrum | 60 (61) | 56 (68) | 2 (14.3) |
| Borderline ASD | 18 (18) | 18 (22) | 1 (7.1) |
| ASD  Borderline + ASD | 20 (20)  38 (38) | 9 (11)  27 (33) | 11 (78.6)  12 (85.7) |

Formulations based on modified DSM-5 classification for ASD

Table 3 Cross-tabulation of clinician formulations of ASD according to vision levels (n=83, 3 missing data) (n, %)

| Vision levels (degree of VI) | Non-spectrum | borderline/ASD | ASD | borderline/ASD plus ASD | Total |
| --- | --- | --- | --- | --- | --- |
| Moderate (plus a few mild) | 33 (79) | 6 (14) | 3 (0.07) | 9 (21) | 42 |
| Severe | 20 (69) | 6 (21) | 3 (10) | 9 (31) | 29 |
| Profound | 1 (11) | 5 (56) | 3 (33) | 8 (88) | 9 |
| Total | 54 | 17 | 9 | 26 | 80 |

Formulations based on modified DSM-5 classification for ASD

Table 4 Principal component analysis of modified ADOS-2®, Module 3 items (2 component model)

| Modified ADOS-2 items | Component 1 | Component 2 |
| --- | --- | --- |
| A5 Offers information | .883 |  |
| A8 Conversation | .847 |  |
| B8 Amount of social overtures/maintenance of attention | .845 |  |
| B4 Shared enjoyment in interaction | .824 |  |
| B10 Amount of reciprocal social communication | .761 |  |
| B7 Quality of social overtures | .752 | .308 |
| B11 Overall quality of rapport | .728 |  |
| B9 Quality of social response | .707 |  |
| C1 Imagination/ creativity | .648 |  |
| B5 Comments on others’ emotions/ empathy | .581 |  |
| A6 Asks for information | .554 |  |
| A7 Reporting events | .541 |  |
| A9 Descriptive, conventional, instrumental and informative gestures | .503 |  |
| A2 Speech abnormalities associated with autism | .465 | .364 |
| B6 Insight into typical social situations and relationships | .439 |  |
| D2 Hand and finger and complex mannerisms |  | .707 |
| A4 Stereotyped/idiosyncratic use of words or phrases |  | .703 |
| D4 Excessive interest in or reference to unusual or highly specific topics or objects or repetitive behaviours |  | .681 |
| D5 Compulsions or rituals |  | .578 |
| A3 Immediate echolalia |  | .560 |
| D1 Unusual sensory interest in play material/ person |  | .512 |

Table 5 ‘Preferred items’ of the modified ADOS-2®, Module 3 for the ‘non-spectrum’ and ‘ASD’ groups (clinician formulation) according to Gotham et al (2007) thresholds

| Item | **‘non-spectrum’ group % (N)** | **‘ASD’ group % (N)** | **Modified/ original (Gotham et al)*** |
| --- | --- | --- | --- |
| *A2. Speech abnormalities Associated with Autism* | 1.8 (1) | 0 (0) | Modified |
| A3. Immediate echolalia | 0 (0) | 77.8 (7) | - |
| A5. Offers information | 1.8 (1) | 44.4 (4) | - |
| *A6. Asks for Information* | 16.1 (9) | 22.2 (2) | Modified |
| *A7. Reporting events* | 25 (14) | 11.1 (1) | Modified |
| A8. Conversation | 0 (0) | 33.3 (3) | Original |
| *A9. Descriptive, Conventional, Instrumental and Informational gestures* | 10.7 (6) | 22.2 (2) | Modified/  Original |
| B4. Shared enjoyment in Interaction | 0 (0) | 55.6 (5) | Original |
| *B5. Comments on other’s emotions/empathy* | 21.4 (12) | 0 (0) | Modified |
| B6. Insight into typical social situations and relationships | 28.6 (16) | 22.2 (2) | - |
| *B7. Quality of social overtures* | 0 (0) | 0 (0) | Modified/  Original |
| B8. Amount of social overtures/ maintenance of attention | 1.8 (1) | 33.3 (3) | - |
| B9. Quality of social response | 0 (0) | 44.4 (4) | Original |
| *B10. Amount of reciprocal social communication* | 0 (0) | 22.2 (2) | Modified/ Original |
| *B11. Overall quality of rapport* | 1.8 (1) | 11.1 (1) | Modified/  Original |
| *C1. Imagination/ Creativity* | 0 (0) | 11.1 (1) | Modified |
| D1. Unusual sensory interest in play material / person | 1.8 (1) | 44.4. (4) | Original |
| D2. Hand and finger and complex mannerisms | 10.7 (6) | 44.4 (4) | Original |
| D3. Self injurious behaviour | 0 (0) | 100 (9) | - |
| *D4. Excessive interest in or reference to unusual or highly specific topics or objects or repetitive behaviours* | 3.6 (2) | 33.3 (3) | Modified/  Original |
| *D5. Compulsions or rituals* | 1.8 (1) | 22.2 (2) | Modified |
| *A4. Stereotyped / idiosyncratic use of Words or Phrases* | 0 (0) | 44.4 (4) | Modified/  Original |

*Items in italics are included in the VI modified algorithm. Right-side column shows whether item is included in Modified or Original algorithms (Gotham et al) or both

Table 6 Principal component analysis (two components) of 12 items in VI modified algorithm

| **12 algorithm items** | **Component (factor)** | |
| --- | --- | --- |
|  | **1 (SA)** | **2 (RRB)** |
| B11. Overall quality of rapport | **.75** | .156 |
| B7. Quality of social overtures | **.73** | .31 |
| C1. Imagination/ creativity | **.71** | .072 |
| B5. Comments on other’s emotions/empathy | **.68** | -.197 |
| B10. Amount of reciprocal social communication | **.66** | .198 |
| A9. Descriptive, conventional, instrumental and informational gestures | **.63** | -.103 |
| A7. Reporting events | **.63** | -.38 |
| A6. Asks for information | **.55** | .081 |
| A2. Speech abnormalities associated with Autism | **.51** | .36 |
| A4. Stereotyped / idiosyncratic use of words or phrases | -.019 | **.72** |
| D5. Compulsions or rituals | .165 | **.71** |
| D4. Excessive interest in or reference to unusual or highly specific topics or objects or repetitive behaviours | .001 | **.67** |

Table 7 Area under curve (AUC), sensitivity and specificity of modified VI algorithms against clinician formulation (‘non-spectrum’ versus ‘ASD’ or ‘borderline-ASD plus ASD’)

| ‘non-spectrum’ v ‘ASD’ categories | AUC | p | Sensitivity | Specificity | Cut-off score | Youden index J | PPV [95% CI] |
| --- | --- | --- | --- | --- | --- | --- | --- |
| Total | 1.00 | p<.001 | 1.00 | 1.00 | 9.5 | 1.00 | 100% |
| SA | .97 | p<.001 | 1.00 | .82 | 6.5 | .82 |  |
| RRB | .91 | p<.001 | .89 | .91 | 1.5 | .80 |  |
| ‘non-spectrum’ v ‘borderline-ASD plus ASD’ |  |  |  |  |  |  |  |
| Total | .90 | p<.001 | .82 | .80 | 7.5 | .62. | 65.62% [95% CI 53.34-77.77] |
| SA | .84 | p<.001 | .82 | .71 | 5.5 | .53 |  |
| RRB | .79 | p<.001 | .63 | .91 | 1.5 | .54 |  |

Table 8 Cross-tabulation of ‘Low Risk for ASD’ and ‘High Risk for ASD’ or ‘High Risk for borderline-ASD plus ASD’, according to vision levels, n=83, 3 missing data (n, %)

| Vision levels | ‘Low Risk for ASD’  (<7.5 cutoff) n | ‘High risk for ASD’ (>9.5 cutoff) | ‘High Risk for borderline- ASD plus ASD’(>7.5) | Total |
| --- | --- | --- | --- | --- |
| Moderate (with some mild) | 31 (74) | 6 (14) | 11 (26) | 42 |
| Severe | 17 (59) | 7 (24) | 12 (41) | 29 |
| Profound | 1 (11) | 4 (44) | 8 (89) | 9 |
| Total | 49 (61) | 17 (21) | 31 (39) | 80 |

Table 9 Comparison of SRS-2 scores between ‘Low Risk for ASD’ (LR) and ‘High Risk for ASD’ (HR) thresholds

| **Subscales** | **Mean Rank**  **LR (n=46)** | **Mean Rank**  **HR (n=14)** |  | | |  |
| --- | --- | --- | --- | --- | --- | --- |
|  |  |  | **U** | **z** | **p** | **Cohen’s d** |
| Social Awareness T-score | 29.08 | 33.27 | 256.50 | -.78 | .44 | .20 |
| Social Cognition T-score | 27.43 | 39.08 | 181.00 | -2.16 | **.04** | **.59** |
| Social Communication T-score | 27.12 | 41.61 | 166.500 | -2.72 | **.01** | **.75** |
| Motivation T-Score | 27.10 | 41.68 | 165.500 | -2.74 | **.01** | **.76** |
| Repetitive Interests T-Score | 28.07 | 36.85 | 210.00 | -1.63 | **.11** | **.43** |
| DSM Social Communication and Interaction T-Score | 26.96 | 40.77 | 159.00 | -2.56 | **.01** | **.71** |
| DSM Repetitive Interests and Behaviours T-Score | 28.07 | 36.85 | 210.00 | -1.63 | **.11** | **.43** |
| Total T-Score | 27.16 | 40.04 | 168.50 | -2.39 | **.02** | **.65** |

Table 10 Comparison of CCC-2 scores between ‘Low Risk for ASD’ (LR) and ‘High Risk for ASD’ (HR) for threshold

| **Subscales** | **Mean Rank**  **LR (n=46)** | **Mean Rank**  **HR (n=14)** |  | | |  |
| --- | --- | --- | --- | --- | --- | --- |
|  |  |  | **U** | **z** | **p** | **Cohen’s d** |
| Speech Scaled | 30.26 | 31.29 | 311.00 | -.19 | .85 | .05 |
| Syntax Scaled | 32.37 | 24.36 | 236.00 | -1.52 | .13 | .40 |
| Semantic Scaled | 29.63 | 33.36 | 282.00 | -.71 | .49 | .18 |
| Coherence Scaled | 31.68 | 26.61 | 267.50 | -.96 | .34 | .25 |
| Inappropriate Initiation Scaled | 31.89 | 25.93 | 258.00 | -1.13 | .26 | .29 |
| Stereotyped Language Scaled | 31.89 | 25.93 | 258.00 | -1.13 | .26 | .29 |
| Use of Context Scaled | 31.72 | 26.50 | 266.00 | -.99 | .32 | .26 |
| Nonverbal Communication Scaled | 31.85 | 26.07 | 260.00 | -1.10 | .27 | .28 |
| Social Relations Scaled | 34.01 | 18.96 | 160.50 | -2.84 | **.01** | **.78** |
| Interests Scaled | 33.51 | 20.61 | 183.50 | -2.45 | **.01** | **.66** |
| General Communication Composite Scaled | 31.63 | 26.79 | 270.00 | -.91 | .36 | .24 |
| Social Interaction Deviance Composite Scaled | 32.46 | 24.07 | 232.00 | -1.58 | .12 | .42 |
